# A Survey and Comparison of Low-Cost Sensing Technologies for Road Traffic Monitoring

**DOI:** 10.3390/s18103243

**Published:** 2018-09-26

**Authors:** Marcin Bernas, Bartłomiej Płaczek, Wojciech Korski, Piotr Loska, Jarosław Smyła, Piotr Szymała

**Affiliations:** 1 Department of Computer Science and Automatics, University of Bielsko-Biala, 43-309 Bielsko-Biala, Poland; marcin.bernas@gmail.com; 2 Institute of Computer Science, University of Silesia, 41-200 Sosnowiec, Poland; 3 Institute of Innovative Technologies EMAG, 40-189 Katowice, Poland; Wojciech.Korski@ibemag.pl (W.K.); Piotr.Loska@ibemag.pl (P.L.); Jaroslaw.Smyla@ibemag.pl (J.S.); Piotr.Szymala@ibemag.pl (P.S.)

**Keywords:** vehicle detection, pedestrian detection, sensor fusion, low-cost sensors, intelligent transport systems, machine learning

## Abstract

This paper reviews low-cost vehicle and pedestrian detection methods and compares their accuracy. The main goal of this survey is to summarize the progress achieved to date and to help identify the sensing technologies that provide high detection accuracy and meet requirements related to cost and ease of installation. Special attention is paid to wireless battery-powered detectors of small dimensions that can be quickly and effortlessly installed alongside traffic lanes (on the side of a road or on a curb) without any additional supporting structures. The comparison of detection methods presented in this paper is based on results of experiments that were conducted with a variety of sensors in a wide range of configurations. During experiments various sensor sets were analyzed. It was shown that the detection accuracy can be significantly improved by fusing data from appropriately selected set of sensors. The experimental results reveal that accurate vehicle detection can be achieved by using sets of passive sensors. Application of active sensors was necessary to obtain satisfactory results in case of pedestrian detection.

## 1. Introduction

The objective of road traffic monitoring is to collect information about different traffic participants. This information is necessary to provide various services that enable smoother, safer, and environmentally friendly transportation. Examples of such services are adaptive traffic signals [1], variable speed limits, traveler information, and route guidance. One of the most important traffic monitoring tasks is detection of vehicles and pedestrians. In case of conventional traffic monitoring systems, this task is carried out with use of intrusive detectors that have to be installed in the pavement (e.g., inductive loops, micro-loop probes, piezoelectric sensors) or detectors that require installation of supporting structures (video detectors, radars). Installation and maintenance of the conventional detectors are expensive and induce serious disruption of traffic.

The disadvantages of conventional detectors have motivated recent development of low-cost sensing technologies for road traffic monitoring that enable easy installation and maintenance of the detectors. This paper reviews the low-cost traffic monitoring methods and compares their accuracy. The comparison of traffic monitoring methods presented in this paper is based on results of experiments that were conducted with a variety of sensors in a wide range of configurations. Main goal of this survey is to summarize the progress achieved to date and to help identify the sensing technologies that provide high detection accuracy and meet requirements related to cost and ease of installation. Special attention is paid in this study to wireless detectors of small dimensions that can be quickly and effortlessly installed alongside traffic lanes (on the side of a road or on a curb) without any additional supporting structures. The wirelesses detectors are powered by batteries. The lifetime of these detectors depends on energy consumption. Therefore, low energy consumption of sensors is also considered as an important requirement. 

Recent surveys of road traffic monitoring systems have focused mainly on vision-based methods [2,3]. Shirazi and Morris [4] reviewed studies on various advanced sensing technologies applied to intersection monitoring. Nellore and Hancke [5] focused on applications of wireless sensor networks in urban traffic management. Pedestrian detection techniques for driving assistance systems have been reviewed in [6]. According to the authors knowledge no comprehensive survey exists specifically addressing low-cost traffic monitoring techniques. This paper provides a complete review and comparison of existing solutions that provide high detection accuracy and meet the above-mentioned requirements related to cost and easiness of installation.

The paper is organized as follows: Section 2 includes a review of literature related to the low-cost traffic monitoring technologies that include applications of infrared and visible light sensors, wireless transmission, accelerometers, magnetometers, ultrasonic and microwave radars as well as acoustic sensing. Vehicle and pedestrian detection experiments are described in Section 3. Details of the experimental results are presented and discussed in Section 4. Finally, conclusions and future research directions are given in Section 5.

## 2. Sensing Technologies

### 2.1. Applications of Infrared and Visible Light Sensors

Results of the research reported in [7] show that a visible light sensor can be successfully applied for detection and tracking of moving objects. Variations of infrared radiation, detected by passive infrared sensor when a vehicle or a person is passing in the vicinity of the sensor, have been utilized in a wake-up system, which allows sensor nodes to be put into sleep mode unless their activation is necessary. Near infrared sensors have been applied for shadows detection of objects [8]. The shadow detection method was designed for video-detection. Possible applications of infrared cameras have been discussed in [9]. However, in this work such applications are not considered due to the high cost of infrared cameras.

Pulsed laser light is utilized in light detection and ranging (LIDAR) technology to measure distances to objects. LIDARs enable speed measurement as well as detection and classification of vehicles [10]. The LIDAR-based solutions are effective but expensive. Moreover, LIDARs should be installed high above the road, which usually requires additional supporting structures.

The sensing technologies based on image analysis (in infrared and visible spectrum) [11], and LIDARs [12] are powerful tools for road traffic monitoring (achieve accuracy above 90%), however their use is associated with high energy consumption.

### 2.2. Methods Based on Signal Strength Analysis in Wireless Communication Networks

In the related literature, several efforts have been made to explore the possibility of road traffic monitoring with use of wireless communication networks. The vehicles detection and localization tasks were performed by analyzing channel state information (CSI) [13], received signal strength indicator (RSSI) [14,15], link quality indicator (LQI), and packet loss rate [16].

A method, which uses wireless transmission to detect road traffic congestion, was proposed in [16]. This method requires wireless transmitters and receivers. The transmitters continuously send packets. The receivers, which are placed on the opposite side of a road, evaluate the RSSI, LQI, and packet loss metrics. It was shown that these metrics enable recognition between free-flow and congested traffic states with high accuracy. The method was implemented and tested with use of ZigBee motes.

A similar ZigBee network was adapted in [15] for vehicle detection. The experimental results presented in that work confirm that a vehicle passing between the network nodes causes a drop of RSSI value. It was also observed that the gradient of RSSI drop depends on the vehicle speed.

In [17] a method was introduced for vehicle detection and speed estimation, which is based on RSSI analysis in network composed of two WiFi access points and two WiFi-equipped laptops. Mean value and variance of RSSI measurements were used to discriminate between three states: empty road, stopped vehicle, and moving vehicle. The experimental results reported in [17] show that variance of RSSI decreases with increasing vehicle speed. This dependency was used for speed estimation.

Another WiFi-based traffic monitoring system was presented in [13]. This system utilizes single access point and one laptop to provide functionalities of vehicle detection, classification, lane identification, and speed estimation. According to that approach, CSI patterns in WiFi network are captured and analyzed to perform the traffic monitoring tasks. The CSI characterizes signal strengths and phases of separate WiFi subcarriers. It was also demonstrated that traffic lanes in a two-lane road have different distributions of CSI data. This fact was utilized to identify in which lane a vehicle is detected.

In [14] a radio-based approach for vehicle detection and classification was introduced, which combines ray tracing simulations, machine learning and RSSI measurements. The authors have suggested that different types of vehicles have specific RSSI fingerprints. This fact was used to perform a machine-based vehicle classification. The RSSI values were analyzed in wireless network of three transmitting and three receiving units, which were positioned on opposite sides of a road. The six wireless units were mounted on delineator posts and equipped with directional antennas. It was demonstrated that such system is able to detect vehicles and categorize them into two classes (passenger car and truck).

The wireless networks have been also used for detection of parked vehicles. In order to detect the parked vehicles, the transmitting nodes are placed on parking surface and the receiving nodes are installed at a high location. When a vehicle is parked over the transmitting node, a decrease of the RSSI value is registered. Thus, the vehicles can be easily detected based on simple RSSI analysis. Different systems of this type were implemented with use of CC1101 wireless communication modules [18] and XBee motes [19].

In [20] Bluetooth low energy (BLE) beacons were used with iBeacon protocol to broadcast data frames. The beacon frames are registered by smartphones that collect the RSSI measurements, aggregate them and send to server for further analysis, which aims at vehicle detection and classification.

The RSSI-based vehicle classification was implemented in the literature with use of various machine learning methods: artificial neural networks, k-Nearest Neighbor (k-NN), support vector machine (SVM), decision trees and logistic regression. A SVM method was adopted in [14] to train vehicle classification models and categorize vehicles into two classes (passenger car and truck). The machine learning algorithms were trained using raw data or a set of predefined features [21].

In [22] a person detection method was introduced, which is based on entropy analysis of registered RSSI values. The experiments were performed with use of radio transmitters and receivers working with a frequency of 2.4 GHz.

### 2.3. Accelerometer Applications

The possibility of vehicle detection and classification based on vibration measurements, with use of accelerometers, has been discussed by several authors in the related literature. In [23] it was shown that detection of the vibrations caused by vehicles is easy to achieve by means of the currently available accelerometers. It is worth to be noted that in this paper the applications are considered of universal (general purpose) accelerometers.

In [24] accelerometers were utilized for impact analysis of vibrations caused by passing vehicles on buildings localized near a road. The measurements were performed in three locations with use of 3185D S/N 2723 accelerometer (Dytran Instruments, Chatsworth, CA, USA) mounted on a 1.2 m long steel bar with diameter of 20 mm. The bar was stuck in the ground to absorb the energy of vibrations. Results of those experiments have shown that personal cars and trucks can be recognized via frequency analysis of the accelerometer readings. 

The possibility of using accelerometers mounted on the road surface for estimating parameters of passing vehicles was analyzed in [25]. The authors of that work have introduced an extended Kalman filter for vehicle tracking based on a moving constant force and a wave propagation model. The experimental results obtained for vehicle tracking in a straight road confirm that such approach enables vehicle detection as well as automatic estimation of vehicle velocity and wheelbase [26]. A disadvantage of that method is dependence of the results on unknown scaling factors.

The authors of [27] have investigated the impact of passing vehicle on measurements performed by accelerometers and magnetometers. The accelerometers are used to detect axle locations of vehicle, while the magnetometers are used to estimate vehicle speed. In the introduced traffic monitoring system, the collected data are synchronized and sent in real time to an access point. The detected vehicles are assigned to one of predefined classes based on calculated axle count and spacing. The authors have concluded that their proposed solution allows the vehicle classification task to be accomplished with accuracy close to 99%.

Experimental and theoretical analysis of the pavement vibrations caused by vehicles was conducted in [28]. The pavement was considered as a wave propagation medium. The authors have estimated parameters of the wave propagation model by using a system identification approach. A model based on the wave propagation theory was proposed and the corresponding parameters have been estimated from measurement data. The introduced model contributes to the understanding of how the road reacts to the load caused by moving vehicles and what kind of vibrations is measured by an accelerometer placed on the pavement.

A sensor network for traffic monitoring with use of multiple accelerometers deployed along and across the road, was presented in [29]. In that solution, high-sensitive piezoelectric accelerometers have been applied that work in micro-g range. An important advantage of this kind of sensors is that they allow the energy of vibrations to be harvested and utilized to power the sensor nodes. For the proposed sensor network dedicated algorithms have been also developed that enable vehicle detection, recognition of driving direction, and speed estimation based on amplitude and frequency analysis of the registered vibrations.

Seismic sensors (three-axis geophone and single-channel seismometer) have been used for vehicle detection by Ghost et al. [30]. The authors of that work have applied time–frequency analysis methods to recognize vibrations produced by passing vehicles. The experiments were conducted on a dirt road as well as on a paved road with asphalt surface. Distance between the sensor and the vehicle detection area was about 3 m. During the experimental evaluation, the impact was considered of disturbances caused by pedestrians and other vehicles. The results presented in [30] show that the proposed solution ensures high accuracy of vehicle detection and enables vehicle classification.

### 2.4. Magnetometer Applications

A method for detection of passing vehicles with use of magnetometers was described in [20]. The most popular solutions based on the magnetic field sensors enable achieving accuracy of vehicles detection comparable to that of the other methods discussed in this Section [31,32]. However, it should be noted that these solutions require the sensors to be installed inside traffic lanes.

Taghvaeeyan and Rajamani [33] have introduced remote magnetic sensors that enable counting and classification of vehicles as well as speed measuring. These sensors can be easily deployed outside traffic lanes without drilling the road surface. The authors have also proposed a vehicle detection algorithm, which uses a model of magnetic field to provide correct results in case of disturbances caused by large vehicles that move in neighboring traffic lanes. The sensors have been connected to create a sensor network. Measurement of vehicle speed in that network was based on evaluating correlation between readings of the sensors, which are placed along the road. Methods of signal analysis in frequency domain have been applied in order to calculate the correlation coefficient in real-time. During experiments the authors have achieved error of speed measurement below 2.5% for vehicle speed in range between 18 and 90 km/h. Classification of the vehicles was based on so-called magnetic length and magnetic height. Length of vehicles was evaluated as the product of speed and time in which a detection zone is occupied by a vehicle. The vehicle height was estimated by taking into account readings of two sensors separated by a distance of 2.5 m. The developed sensor network has enabled detection of turning vehicles at a crossroad with accuracy of 95%.

In another work along these lines [34] a sensor network was proposed, which enables counting and classification of vehicles. A sensor node was designed, which includes a magnetometer, accelerometer, temperature sensors, GPS module and an IEEE 802.15.4 wireless communication interface. The task of single sensor node is to detect and count vehicles in real-time. Detection algorithms were developed that take into account compensation of temperature changes. The GPS module and T-sync algorithms were utilized to synchronize the sensor nodes. This approach has enabled the sensor nodes to be synchronized with nanosecond accuracy. Experimental results have revealed that the proposed sensor network can achieve vehicle detection error below 1%. The accuracy of speed measurement was equal to 96% (RMSE below 10 km/h). Production cost of the sensor node was estimated at 40$.

Portable anisotropic magneto-resistive sensors were applied for traffic monitoring by Jinturkar and Pawar [35]. During experiments a sensor network with four sensor nodes installed in road-side, by a traffic lane was tested. The authors demonstrated that their sensor network allows vehicle classification to be performed based on mean magnetic height and width. Speed of vehicles was estimated by taking into account readings delivered by two neighboring sensor nodes. The wireless communication between nodes was established by using Zigbee modules. The presented experimental results confirm that the magnetic sensors enable precise road traffic measurements in variable weather conditions. 

More complex architecture of sensor node for traffic monitoring was proposed in [36]. The sensor node was equipped with an ATxmega128A4 microcontroller (Microchip Technology Inc., Chandler, AZ, USA) and a set of sensors, i.e.,: magnetometer, accelerometer, humidity sensor, thermometer, barometer, rain sensor, microphone, and light sensor. For experimental evaluation, the sensor nodes were installed inside traffic lanes. The data collected by magnetometer were utilized for vehicle detection and classification. During tests nine classes of vehicles have been successfully recognized based on their magnetic signature. Moreover, additional tests were performed with sensor node placed in road-side. The results have revealed that in case of sensor nodes located outside traffic lanes, the measurements of magnetic signature are significantly distorted, which makes it difficult to classify the vehicles with high precision. For the sensors installed outside traffic lane, it was also observed that small vehicles (e.g., motorcycles) may be undetected. The authors have also analyzed the impact of magnetometer orientation on the results of vehicle detection and classification. It should be noted that in the afore-mentioned solution, the sensor nodes were synchronized by using real-time clock and GPS module.

The wireless nodes with magnetic sensors have been also applied for parking vehicle detection in on-street parking [37]. The authors of that work have proposed vehicle detection and speed measurement algorithms that are based on correlation analysis of sensor readings collected by neighboring nodes. The experimental results have confirmed that the proposed solution enables detection of parking vehicles and speed estimation with accuracy of 99% and 92%, respectively. 

### 2.5. Ultrasonic and Microwave Radars

Radars are commonly used for vehicle detection. A vehicle detection method with use of ultrasonic sensors was presented in [38]. Sensors of this kind are also used in vehicles for development of on-board systems that enable detection of obstacles [39].

In [40] a micro-radar was presented for detection of bicycles. That micro-radar is installed in the pavement, emits a high-frequency radio signal with low energy and measures the energy reflected from a passing objects. A profile of the reflected radio signal is analyzed. On this basis, the sensor estimates size of passing objects and recognizes bicycles.

### 2.6. Acoustic Sensing

A vehicle detection system based on acoustic sensors was proposed in [41]. That system uses a sensor unit, which consists in a pair of microphones deployed along the roadside. The detection method utilizes the fact that sound of passing vehicle reaches the two microphones at slightly different times. The time difference is estimated by computing cross-correlation of the microphone readings. Peak of the cross-correlation function corresponds to position of the vehicle. Thus, that approach enables determination of so-called sound map, which represents the vehicle motion along a predefined track. Details of this method are discussed in [42,43].

In [44] a method was presented for sound pattern classification, which enables sound recognition of heavy, medium, and light vehicles as well as horn. The underlying approaches to sound patterns acquisition and separation are presented in [45].

Design and implementation of another acoustic system for road traffic monitoring was described in [46]. That system utilizes a grid of 37 microphones. It allows the measurement of traffic parameters to be performed for multiple traffic lanes. Centers and edges of particular traffic lanes in the monitored area are recognized automatically. The measurements include vehicle counting and classification as well as estimation of average speed and lane occupancy. Two vehicle classes (long and short) were considered during testes of the system. The class of vehicle was recognized by taking into account occupancy time of a detection area. The test experiments have been conducted on a road with three traffic lanes. Counts of vehicles were determined with accuracy of 85%, however the error of vehicle classification have reached 90% for the long vehicles. The authors of that work have suggested that better results could be obtained by using more sophisticated classifiers, e.g., decision trees or support vector machines.

George et al. [47] have introduced a method for vehicle detection and classification on the basis of acoustic signal registered by a single microphone. In that method the vehicles are recognized with use of k-NN and artificial neural networks classifiers. Input data of these classifiers include coefficients that describe the acoustic signal. A preprocessing procedure was implemented to reduce noise and detect peaks of the analyzed signal. The method has enabled recognition of three vehicle classes, i.e., heavy, medium, and light vehicles.

### 2.7. Sensor Fusion

Sensor fusion methods are used in several traffic monitoring approaches to combine data from multiple sensors of different type in order to detect vehicles and recognize their characteristics (speed, class, etc.). 

Data collected from accelerometers and magnetometers have been utilized for vehicle classification in [27,48]. Wenteng et al. [27] have introduced a prototype of vehicle classification system, which recognizes axle count and spacing. In that system, the accelerometers detect vehicle axles, while the magnetometers report vehicle arrivals and departures and estimate their speed. The vehicles were categorized into three classes (2-axle car, 3-axle car, and 5-axle heavy truck). Test results have shown that the prototype is reliable in classifying vehicles with 99% accuracy.

A method for determining vehicle speed and position based on fused measurements of magnetometers and accelerometers was presented in [48]. The authors have shown that the measurements of the two different sensors can be fused using the particle filtering approach. The performance of that method was verified by computer simulations. The results were compared with accuracy of particle filters that process only measurements of one of the sensors. It was found that the vehicle tracking system performs better when data from the two sensors are taken into account.

Fernández-Lozano et al. have presented a wireless sensor network, which utilizes data obtained from Bluetooth nodes as well as from ultrasound and laser sensors to collect information about trip origins and destinations for particular vehicles in a given urban area. The Bluetooth nodes enable detection of Bluetooth devices installed in vehicles. According to that approach, the vehicles are identified based on MAC addresses of the built-in Bluetooth devices. The ultrasound sensors were implemented to detect the number of vehicles passing through the ultrasound beams. The vehicles detection and counting functions are also supported by the data obtained from the laser sensors. Accuracy of that system was demonstrated through experiments in real traffic conditions.

### 2.8. Selection of Sensing Technologies

Based on the above literature survey, the usefulness of sensing technologies was evaluated with regard to the cost, dimensions, energy, and portability requirements. Table 1 includes summarized information about sensing technologies that are potentially useful in development of traffic monitoring systems. The sensors that fulfill all considered requirements were used during experiments to compare their performance in traffic monitoring applications. The experiments and their results are described in the next sections. Table 2 shows the representative works for the selected technologies regarding their goals, features, applications, and important findings. The most important advantages and limitations of these technologies are summarized in Table 3.

## 3. Experiments

**Figure 1:**
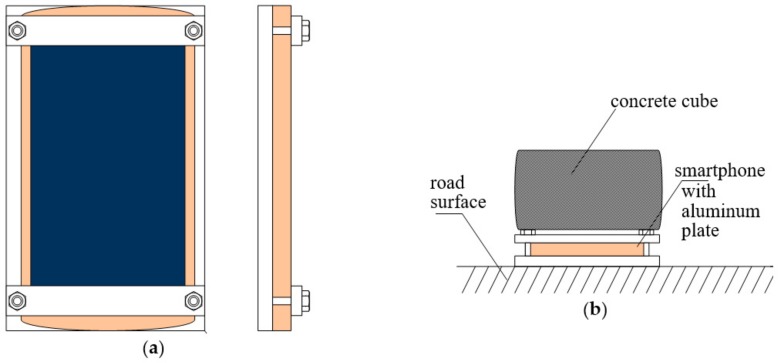
First model of sensor node (SN1): (**a**) smartphone attached to aluminum plate; (**b**) placement of the device during experiments.

Extensive experiments were conducted to compare effectiveness of different low-cost sensing technologies in traffic-monitoring applications. During experiments, two different models of sensor node were utilized. An off-the-shelf smartphone (Redmi 3s, Xiaomi, Beijing, China) was adapted to implement the first model of sensor node (SN1), as presented in Figure 1. In order to collect the data from built-in sensors of SN1, a mobile application was developed, which sends these data to server for further analysis. The sensors available in SN1 are listed in Table 4. Additionally, the GPS module of SN1 was used to precisely determine the time when sensor readings were collected. This function enables synchronization of the datasets registered with use of multiple sensor nodes.

**Figure 2:**
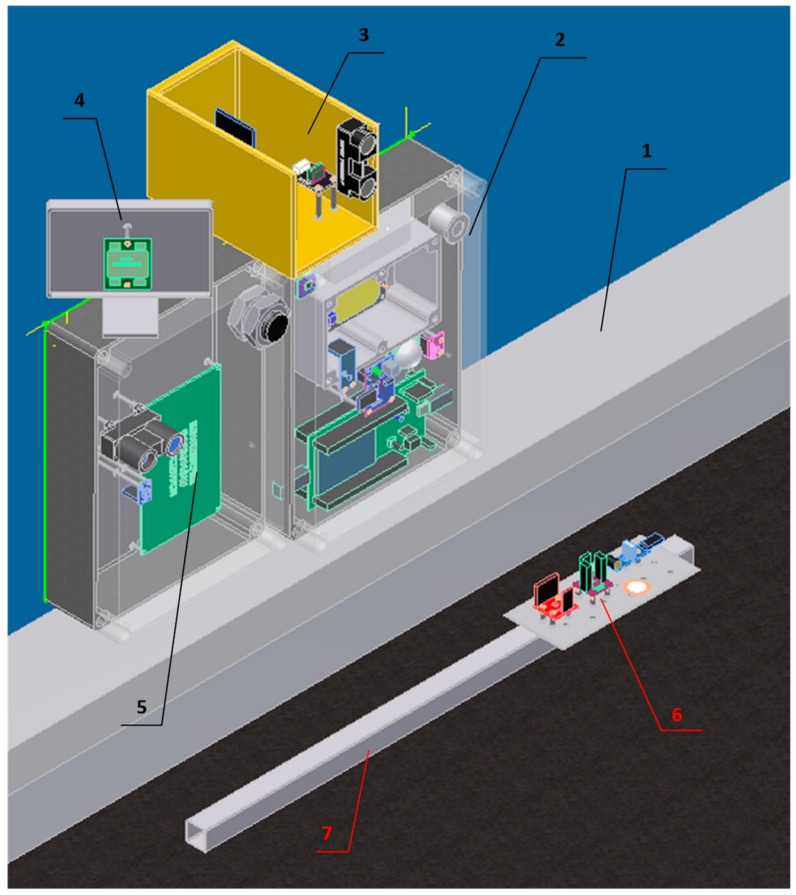
Second model of sensor node (SN2): (1) curb, (2) main box with microcontroller and sensors, (3) ultrasonic sensor, (4) Doppler radar, (5) LIDAR, infrared sensor, and infrared camera, (6) accelerometers, (7) aluminum profile glued to the road surface.

An extended set of sensors was installed in the second model of sensor node (SN2). The design of SN2 is shown in Figure 2 and the installed sensors are listed in Table 5. The sensor type was selected using a low cost threshold (10$ in case of passive sensors and 50$ active ones), low-cost sensors that offer the best sensitivity were purchased. Therefore, some expensive solutions, e.g., directional microphones, high-end accelerometers and seismic sensors, were excluded from the analysis. The presented construction of sensor node was based on results of preliminary experiments. During the preliminary research various methods of sensor installation were considered. The most promising options have been selected for the design of SN2.

**Figure 3:**
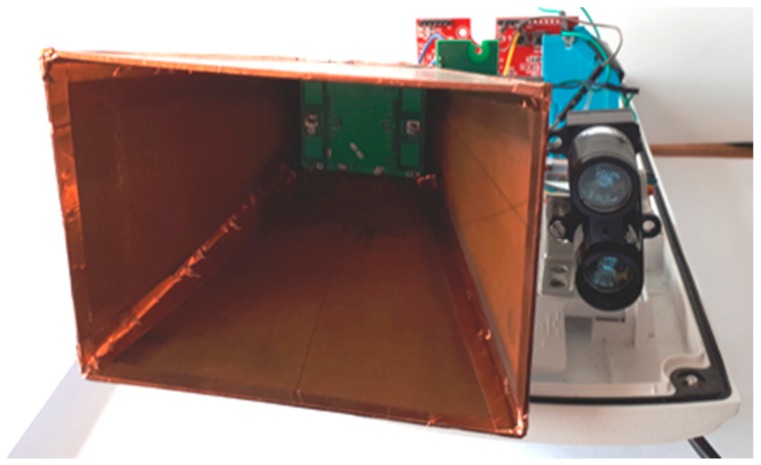
Housing of Microwave Doppler radar HB100.

The microwave radars (7644 HB100 and SEN0192) were placed in simple metal housings to reduce the impact of objects or persons behind the node on sensor readings. Radar SEN0192 was directed perpendicular to the axis of the road, while radar 7644 HB100 was oriented 45 degrees to this axis. Despite using simple housing, the influence of an environment was still high, thus a dedicated cover was designed for microwave radar HB100. The construction was presented in Figure 3. The dedicated housing allows one to reduce the influence of background on the sensor readings and set direction of the microwave beam. During experiments, it was noticed that the stable shield on the opposite road side increases active sensors performance. Additionally, the angle between a metal object (e.g., vehicle) and the sensor beam also influences the measurements.

**Figure 4:**
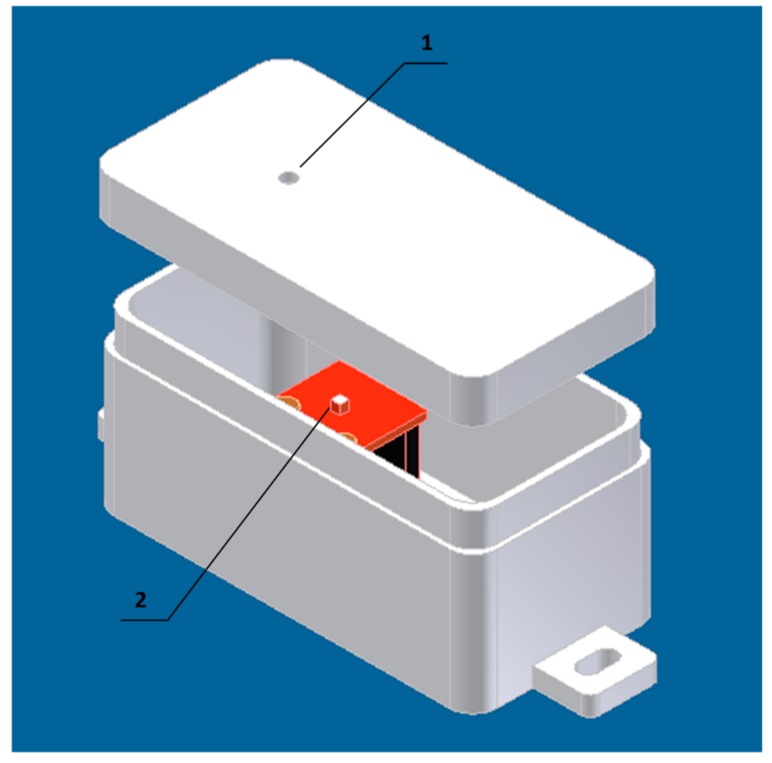
Housing of light sensor: (1) hole with diameter 3 mm, (2) sensor ISL2915.

Additional housing was also designed for the light sensor ISL2915 (Figure 4). A small hole was drilled in the opaque housing to adjust the direction of light rays that are detected by the sensor. This solution makes the sensor more sensitive to the changes of light intensity that are caused by passing vehicles. The preliminary tests have shown that without the proposed housing, the vehicle detection based on light sensor is impossible in bright sunlight. 

Initial experiments were also performed to select appropriate installation method for vibration sensors (accelerometers). These experiments have revealed that the measurement of vibrations on road surface is more effective than the application of a bar stuck in the ground. Such observation is consistent with the result reported in related literature. Therefore, the accelerometers were mounted on a steel plate bolted to an aluminum profile (5 m long, cross section of 20 × 20 mm). The profile was glued to the surface of the road, as shown in Figure 2.

During experiments two SN1 nodes were placed on opposite sides of a traffic lane. Distance between the nodes was of 3.5 m. Sensor readings were collected from one of the nodes in time intervals of 200 ms. The second node was used as a transmitter for the RSSI measurement. The time of car detection events was registered by human observers, when vehicles were passing between the sensor nodes. The events were also registered when no vehicle was present in vicinity of the sensor nodes. A dedicated mobile application was used by the observers to collect the events.

Similar experiments were performed with a single SN2 node. The SN2 node was placed on the curb, as presented in Figure 2. In this case a car was parked on the opposite side of the road (in distance of 5.5 m from SN2) to obtain stable reference values of the measurements made by the active sensors (LIDAR, radar). The objective was to detect the vehicles passing between the SN2 node and the parked car (shield). The measurement data from SN2 were acquired with different frequencies for particular sensors. These frequencies were set according to specifications of the sensors.

**Figure 5:**
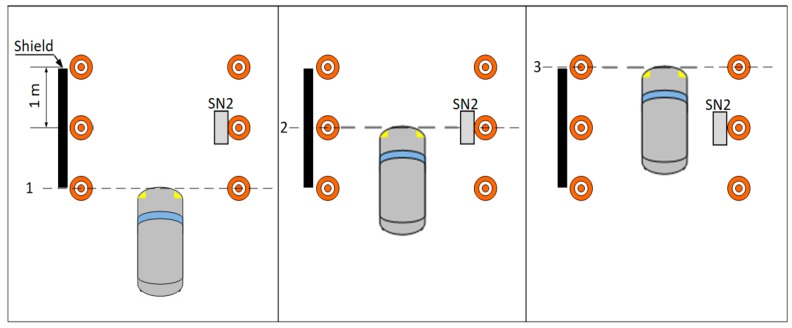
Schema of vehicle localization experiments.

Next part of the experiments with SN2 was devoted to vehicle localization in a detection area with accuracy of 1 m. During these experiments the human observers have registered time of three events: (1) vehicle enters detection area, (2) vehicle occupies half of the detection area, and (3) vehicle reaches end of the detection area. Length o the detection area was 2 m. A schema of the localization experiments is presented in Figure 5. The reference data, describing the aforementioned events, were collected by two human observers. If an event was not confirmed by two observers, then such event was ignored. It should be also noted here that all the above discussed test scenarios for SN1 and SN2 were also used to examine the possibility of pedestrian detection.

The objective of localization experiments was to explore the possibility of localizing vehicle or pedestrian with use of the low-cost sensors that are originally designed for vehicle or pedestrian detection. During these experiments it was verified if the sensors installed on road side, which detects vehicles/pedestrians, can additionally recognize position of the detected target within detection area. Thus, the localization is considered in this study as a supplementary function of the detectors. It should be also noted here that vast amount of dedicated localization methods is available in the literature. Some of them use the same sensor types as considered in this study but in different configurations (e.g., radars installed on vehicle [49]). These dedicated localization methods are out of the scope of this study.

The data registered during measurements by each sensor in SN1 and SN2 were aggregated with use of so-called sliding window [50]. According to this method, if a new sensor reading is registered at time *t*, then the aggregation is performed on a set of data readings for which the registration time *t’* satisfies condition *t* − *w* ≤ *t*’ ≤ *t*, where *w* is size of the time window. Such set of sensor readings is used to calculate aggregates (statistics) of the measured values, i.e., minimum, maximum, median, average, and standard deviation. During initial research it was noticed that by increasing the window size up to 1 s the detection accuracy was increased. However, if the window is wider than 1 s, the measurements registered for two successive vehicles can be aggregated and the detection accuracy decreases significantly. Thus, the window size (*w*) of 1 s was used for further experiments.

The presence of an object (vehicle or pedestrian) in the detection area was recognized based on the aggregated data, by using different machine learning methods, i.e., decision trees (DT), k-nearest neighbors algorithm (KNN), and multi-layer perceptron neural network (MLP). The machine learning methods were selected with regard to the computational requirements and possibility of implementation in cheap, energy-efficient software platforms.

The KNIME software with Weka tools [51,52] was used for classification purposes. The DT classifier uses a C4.5 algorithm (gain ratio was used as quality measure without pruning) thus no additional parameters has to be tuned. In case of MLP classifier the RPROP implementation of the multilayer feed-forward networks was used [51], which performs a local adaptation of the weight-updates according to the behavior of the error function. Various structures of MLP were considered with one and two hidden layers. The number of hidden neurons was between 10 and 20. Each neuron had sigmoid activation function. The MLP structure with one hidden layer and 12 hidden neurons was selected, based on results of cross validation obtained for 100 iterations. Finally, the k-NN algorithm without weighting and linear search was used. Thus, only one parameter, i.e., the number o neighbors (*k*) was tuned. Using the cross validation method, the optimal value of *k* parameter was selected (*k* = 3). Sensor data collected for 60 events (passing vehicles/pedestrians) were used for training of the machine learning algorithms. The data used for parameter selection was excluded from further evaluation process.

The machine learning algorithms were also applied for the localization problem to recognize three locations of a vehicle/pedestrian, as shown in Figure 5.

During experiments, the machine learning algorithms were fed with three sets of the data aggregates. The first set of input data contained minimum, maximum, and median values. The second set consisted of average and standard deviation. In the third case, the values of minimum, maximum, average, and standard deviation are used. In order to evaluate accuracy of object detection, the aggregated data from sensor nodes were divided into training dataset and test dataset in the proportion of 60% to 40%. The detection (localization) accuracy was determined using the following formula:

(1) $$\mathrm{Accuracy}=\frac{\sum_{i=1}^{n}C_{i}}{S}$$

where: *n*—number of classes, *C_i_*—number of items in the test dataset that are correctly assigned to *i*-th class, and *D*—number of items in test dataset. It should be noted here that two classes (*n* = 2) were taken into account for the detection problem: (1) object present in the detection area, (2) no object in the detection area. In case of the localization problem, four classes were considered (*n* = 4): (1) object enters detection area, (2) object present in the detection area, (3) object reaches end of the detection area, (4) none of the above. The use of the accuracy measure was motivated by the fact that the prepared test dataset includes equal number of elements for each considered class.

## 4. Results and Discussion

The sensor data collected by SN1 were used to evaluate the accuracy of vehicle and pedestrian detection. Table 6 shows the results that were obtained based on individual sensor readings with use of DT algorithm. Input data of the algorithm have included four aggregates: minimum, maximum, average, and standard deviation. The measurements collected by accelerometer, gyroscope and magnetometer were analyzed separately for the three axes, while the remaining sensors provided single value. It should be noted here that the accuracy values close to 50% can be obtained as random results.

During experiments with the standard off-the-shelf device (SN1) the high vehicle detection accuracy was achieved by using microphone, wireless communication (RSSI), and magnetometer. The accelerometer readings of SN1, due to sensor quality and type of the connection with surface, were insufficient to correctly recognize the vibrations. The vibrations were sufficiently detected only if a pedestrian was walking very closely to the sensor node (changes of sensor readings were observed for x and y axis). The data from gyroscope were characterized by strong noise, thus the results of classification were significantly affected. This effect can be observed for pedestrian run, where the accuracy values for x and z axes are quite different. Magnetometer readings are not affected by non-magnetic objects. Therefore, the magnetometer has provided accurate detection only for vehicles. In this case the accuracy is above 80%, while accuracy of pedestrian detection is close to 50%. The best results were obtained for x and z axis, thus at least those two should be considered to detect vehicles. The fact that magnetometer readings are unaffected by pedestrians enables distinguishing pedestrians from vehicles, when collecting data from several sensors.

Accurate results for both vehicles and pedestrians (accuracy over 90%) were obtained only with use of a microphone. The sound of car as well as walking and running person was detected using standard microphones installed in SM1. The sound of engines and running persons was easier to detect than the sound of a walking person. Thus, 8% decrease of accuracy was noticed when comparing the results obtained for walking person with those for running person. Finally, it should be noted that the microphone readings can be affected by sound sources other than the objects to be detected, e.g., vehicles in neighboring traffic lanes. During measurements discussed in this Section, the other sound sources were not present in vicinity of the sensor node.

The light sensor in SN1 was affected by sun reflections from vehicles and shadows of trees, thus its accuracy for vehicle and running pedestrian detection was relatively low. Only in case of slowly moving object (walking pedestrian) the light sensor was sufficient to detect the object in most cases (79% accuracy). Therefore, in SN2 the special housing was proposed and the data collection interval was decreased to 100 ms for this sensor type.

The detection results obtained with use of RSSI data shows that the RSSI sampling rate of 5 Hz allows us to detect vehicles with fair accuracy (85%). However, during experiments it was also observed that the effectiveness of RSSI-based detection strongly depends on sampling rate and height above road surface, at which the devices are installed. The detailed research results related to these aspects were presented by the authors in [20].

**Figure 6:**
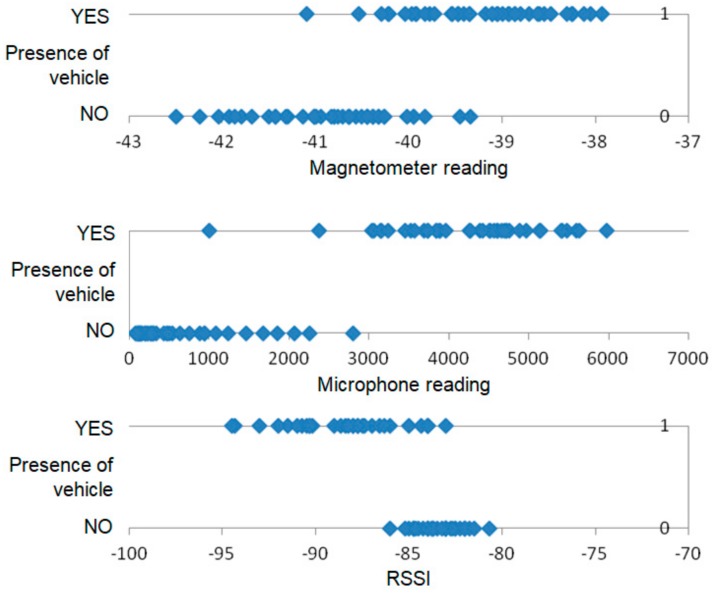
Comparison of measurements collected by SN1 for vehicle presence and absence.

**Figure 7:**
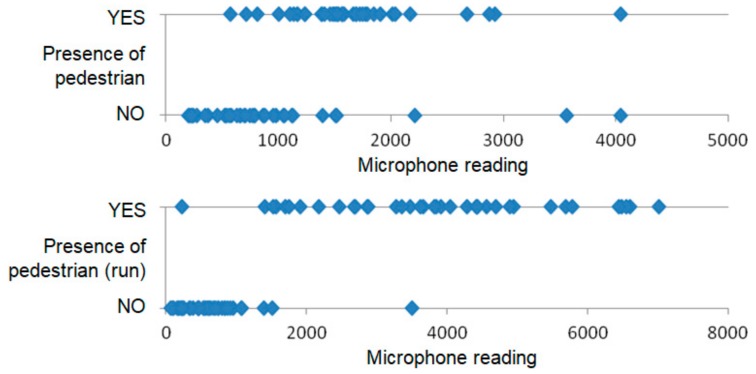
Comparison measurements collected by SN1 for pedestrian presence and absence.

A subset of the data collected during the experiments with SN1 is depicted by the scatter plots in Figure 6 and Figure 7. The values presented in that scatter plots are average sensor readings determined for sliding window of 1 s. These charts allow us to compare the dispersion of measurement results for sensors that have provided high accuracy of detection. Additionally, the accelerometer was considered, as one of the sensors that have been used for vehicle detection in previous works.

The above discussed results show that for individual sensors there is no clear separation between the readings registered when a vehicle is present in the detection area and those collected in case of empty detection area, thus the data from multiple sensors should be considered.

**Figure 8:**
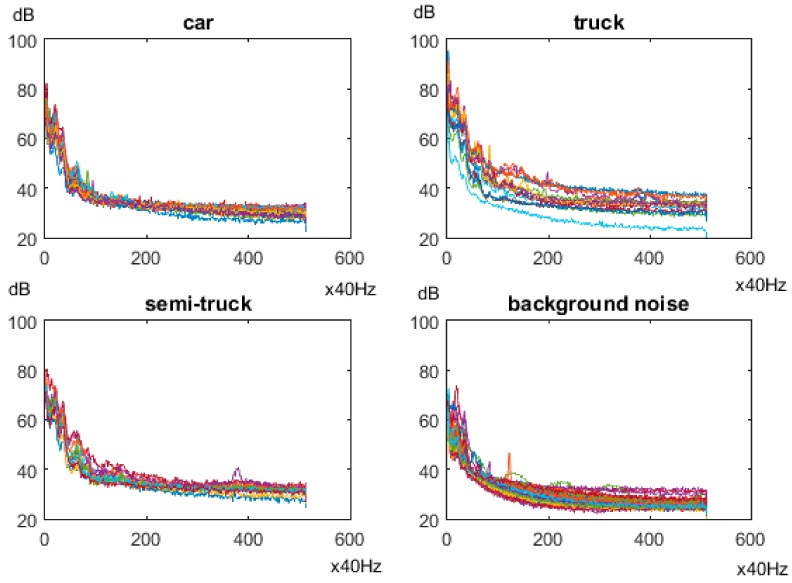
Sound spectra for vehicles of different classes and for background noise.

SN1 was also used to measure sound level in a four-lane road with high traffic intensity. In this case a significant background noise was caused by the vehicles in neighboring traffic lanes. The measurements were analyzed in frequency domain to verify the possibility of detecting vehicles. The objective was to recognize the vehicles passing in the right-most traffic lane (SN1 was placed on the right side of the road). Sound spectra for three vehicle classes and for background noise are presented in Figure 8. Each chart in Figure 8 shows the spectra for 20 samples registered in real traffic conditions. The sound spectra for vehicles of the same class as well as for the background vary significantly. Thus, it is impossible to distinguish between vehicle and background for a large part of the analyzed samples. These results show that the accurate vehicle detection with use of microphones requires more complex data, e.g., sound levels measured by microphones placed in different lanes.

As mentioned above, the experiments with SN1 nodes were also performed in urban road during periods of high traffic intensity, when time headways between vehicles were small and other vehicles were passing in adjacent traffic lane. It was observed that for such conditions higher detection accuracy can be achieved by decreasing the size of the aggregation window. Further improvement would be also possible after implementation of the methods from the literature that deals with the problem of multiple vehicles present in the detection area. Different methods are available for particular sensor types, e.g., for microphones [44] and magnetometers [33]. In this study such methods were not applied as the objective was to compare the effectiveness of different sensors in similar settings, using algorithms that have low computational complexity and can be implemented in cheap, energy-efficient hardware platforms.

Further tests were performed to check the possibility of improving the vehicle detection accuracy by combining the data collected from different sensors available in SN1. Two datasets were taken into account, selected based on the results presented in Table 6. The first (full) dataset consists of data readings from magnetometer (for three axes), microphone, and RSSI values. The second dataset contains magnetometer data (for axes axes) and RSSI values. The microphone readings were not considered in this dataset as they can be affected by presence of other strong sound sources, as explained above.

The vehicle detection accuracy for the two datasets is compared in Table 7. The results in Table 7 are presented for two different sets of the aggregates and three classification algorithms. It should be noted that the sets of aggregates considered in this case include less elements than the set, which was used to collect the result presented in Table 6. The set of aggregates was selected as giving the highest accuracy values. In majority of cases, with exception of MLP algorithm and reduced dataset, the average and standard deviation aggregates provided better or the same results as median, minimum and maximum aggregates. An important observation is that 100% accuracy of the detection can be achieved by taking into account the three sensors (magnetometer, microphone, and wireless module measuring RSSI). Thus, the sensor fusion has enabled improvement of the detection accuracy in comparison with the results achieved for individual sensors. The improvement was possible despite the fact that the set of aggregates was reduced. The worse result of MLP algorithm could be caused by the fixed neural network size, selected during initial research, as well as by limitations of the training procedure, which was used to adjust the weights of inter-neuron connections. In case of MLP algorithm both the network structure parameters as well as the weights have to be tuned. The large number of parameters and low number of iterations can give semi-optimal result. Therefore, 10 training sessions were conducted and average results are presented in this Section. The DT and KNN algorithms have achieved 100% accuracy. It should be noted that those two algorithms do not have to use all input data for decision making, thus they are less affected by outliers. In practice the DT algorithm can make the decision based on single attribute, if the selected attribute is sufficient. Additional advantage of DT over KNN is lower memory utilization. The DT in contrast to KNN does not have to store the training database in the memory.

A decrease of detection accuracy was experienced when taking into account the second dataset without microphone readings. In this case the accuracy was only slightly better than that achieved for individual sensor (magnetometer). When comparing the considered machine learning algorithms, it can be observed that MLP gives worse results than DT and KNN. The reduction of input dataset has influenced the results of DT algorithm. Nevertheless, the DT algorithm managed to retain the high detection accuracy (88%).

Another observation is that two aggregates, i.e., average and standard deviation, were sufficient to accurately detect vehicles. The accuracy achieved for the experiments without sound varies in range of 3% for the considered sets of aggregates. All three compared algorithms have their advantages. The decision-making procedures of MLP and DT algorithms can be implemented in sensor node, due to low computational complexity and memory requirements. In contrast, the KNN algorithm has significantly higher requirements related to computations and memory resources. Therefore, for further tests the DT algorithm was used as giving the most stable results (unaffected by parameters tuning).

**Figure 9:**
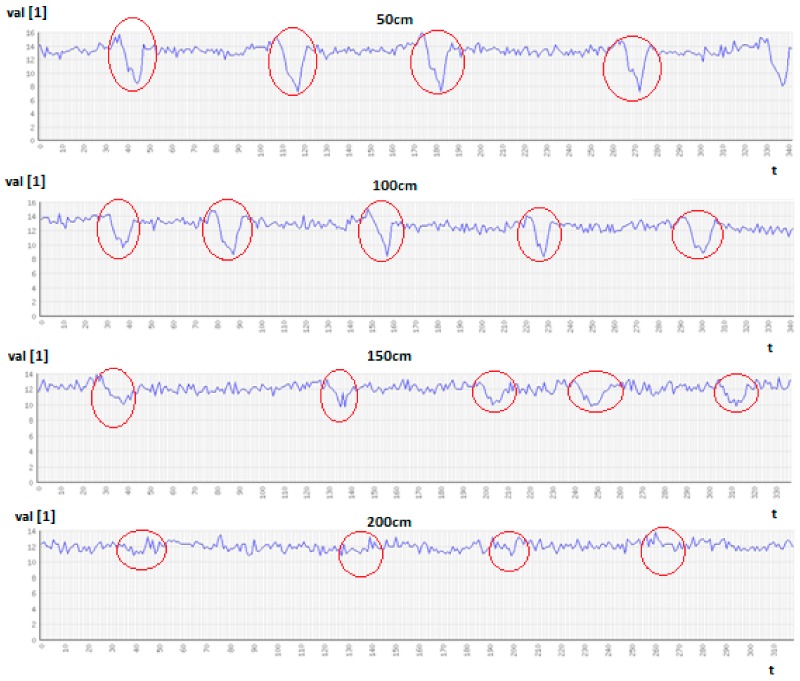
Readings of magnetometer for various distances between vehicle and sensor.

Similar research and analysis were conducted using the SN2 node. Results of vehicle and pedestrian detection based on data delivered by individual sensors in SN2 are presented in Table 8. The detection and localization tasks were performed with use of DT algorithm. Input datasets of the DT algorithm have consisted of minimum, maximum, average, and standard deviation values determined for sensor readings within sliding window. Results of these experiments show that vehicles were accurately detected with use of magnetometer and light sensor. For magnetometer the high accuracy is achieved provided that the vehicles pass close to the sensor location. The detection accuracy drops significantly if the distance between magnetometer and vehicle is above 2 m. Examples of magnetometer readings are presented in Figure 9. The time intervals, during which a car was close to the sensor, are marked as red circles. Minimum distances between the car and the sensor are presented above the charts.

In case of magnetometer, the detection accuracy decreases with increasing distance between the sensor and vehicles (see Figure 9). A similar effect was observed for the majority of the passive sensors, including the light sensor and accelerometer. Thus, the placement of passive sensors is a key factor. Light sensors achieve a high detection accuracy during sunny days, under stable ambient lighting conditions. It should be noted that the results were improved in comparison with those of SN1 by using the housing presented in Figure 4, which allows the light sensor to be appropriately oriented. For such conditions vehicles and pedestrians can be detected in distance of 4 m. In such settings, the detection accuracy for light sensor was increased to 95%. However, in case of dynamic lighting changes, e.g., during cloudy weather, the detection accuracy is significantly lower (equals 60%). Nevertheless, the analysis of light sensor readings can help in detecting the changes generated by environment and those related to moving object close to the sensor. 

**Figure 10:**
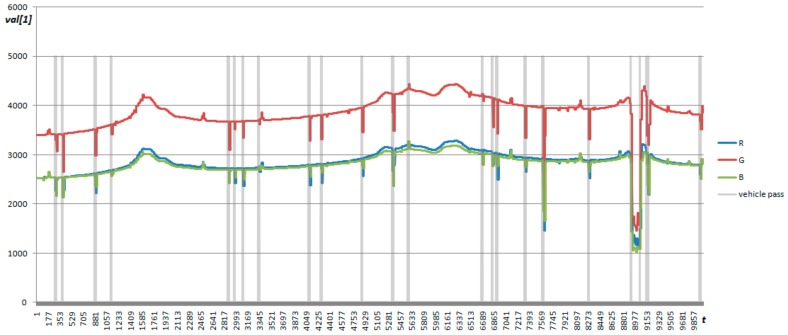
Readings of light sensor.

An example of the signal registered by light sensor is presented in Figure 10. The changes of light value in case of passing vehicle are more rapid than in background. The color of a vehicle also influences the light sensor readings: the registered value can decrease or increase when vehicle is present in front of the sensor.

During experiments, the accelerometers enabled accurate detection of heavy vehicles (trucks). However, personal cars were not correctly detected using this type of sensor. The results achieved with use of the LSM9DS1 accelerometer are better than those obtained for the remaining accelerometers installed in SN2. The accuracy of accelerometer-based detection strongly depends on sensor sensitivity, type of the road, and type of the sensor mounting. In comparison to the results presented in [25], the sensitivity of the analyzed sensors was lower. Thus, a more precise accelerometer can be used to improve the detection accuracy. Based on the literature [23], it was expected that the piezoelectric sensor will provide useful data for vehicle/pedestrian detection. However, the experiments did not confirm that assumption. The low detection accuracy observed for the piezoelectric sensor could be caused by the type of its installation. The results can be also improved for this sensor by using a pre-amplifier.

The LIDAR proved to be useful sensor for vehicle detection, provided that a shield is used, as shown in Figure 5. LIDAR could be also installed over the traffic lanes at some height. However, without an object (or shield) present in sensing range it losses stability and becomes ineffective. During research in real-traffic conditions it was noticed that narrow LIDAR detection beam could be avoided by vehicles if the traffic lane is wide.

The research has also covered the test of barometer, which proved to be effective if distance to the target object is short—up to 0.5 m. However, the barometer loses its applicability in case of strong wind or when closed in a cover. Finally, the microwave radars are able to detect moving object, however without a dedicated design of housing they collect the noise from surroundings. Therefore, a special housing was proposed to improve the accuracy of microwave radar (Figure 3). The results obtained with use of the proposed housing show improvement, in case of both vehicles and pedestrians detection.

Comments related to each type of the sensors are summarized in Table 8. A general observation is that high detection accuracy cannot be achieved with use of individual sensors for all considered situations.

Before selecting subsets of sensors for further tests, the relation between their data readings was examined with use of Pearson’s correlation coefficient. The readings for X and Z axis of the magnetometer showed strong correlation (−0.98), thus in applications only one axis can be considered. Similar relation was observed for R, G, B values registered by the light sensor (0.97). The high correlation was also observed for accelerometer and light sensor (0.8). Finally, weak correlation was noticed between light sensor, accelerometer and LIDAR readings, where the correlation coefficient equals 0.85 and 0.91, respectively.

Despite the aforementioned dependencies, the full set of sensor readings was initially used for detection of objects. Table 9 presents the accuracy of vehicle detection and localization, which was obtained by using different subsets of the sensors available in SN2. The results of pedestrian detection and localization are shown in Table 10. In Table 9 and Table 10 three categories of sensor sets are distinguished: sets containing only passive sensors, sets of active sensors, and sets that comprise both passive and active sensors. For the passive and active sensor categories full set of the considered sensors is presented in the first row. The detection accuracy was tested using all possible subsets of the sensors. The subsets that have provided high detection and localization accuracy are included in Table 9 and Table 10. In case of the last category, which combines passive sensors with active sensors, the results are presented for the subset that contains the lowest number of sensors and achieves the highest accuracy.

The results in Table 9 and Table 10 clearly show that high detection accuracy can be achieved by using the active sensors as well as the passive sensors. Vehicles were correctly detected (98% accuracy) based on data from three sensors: magnetometer, accelerometer, and light sensor. The same result was obtained in case of magnetometer and light sensor. However, the localization accuracy was decreased in that case. Addition of the other sensors did not improve the accuracy of vehicle detection. The best results regarding vehicle localization (in the passive sensors category) were observed for the sensor set consisting of magnetometer, accelerometer and vibration sensor. However, these results are not satisfactory, since the localization accuracy did not exceed 72%. The combination of passive and active sensors has improved these results only by 1%. This value shows that the range of passive sensors is limited as mentioned above.

In case of pedestrian detection (Table 10) the active sensors performed better than the passive sensors. The best result (95% accuracy) was obtained by using microwave radar and LIDAR. For passive sensor set (accelerometer, light sensor, and PIR) the detection accuracy reached 89%. The results show that correct localization of pedestrians cannot be obtained when using only one sensor node. Therefore, for localization purposes of pedestrians and vehicles a sensor network with multiple sensor nodes installed along the road would be recommended. It should be also kept in mind that dedicated localization methods, which can provide better accuracy, were not considered in this study, as it is focused on the portable, easy to install, low-cost solutions.

The experimental results show that type of the housing as well as position of sensor influences the readings and detection accuracy. Data readings of LIDAR, light sensor or PIR are influenced by the housing and its orientation. The barometer was useful, while installed without any cover.

The RSSI value depends strongly on sensor position: it increases when sensors are installed over 50 cm above pavement and decreases when sensors are situated on the ground. It was also observed that the magnetometer readings were useful if a distance to the detected object was lower than 2 m. Finally, the accelerometer has to be firmly glued with the surface to provide useful measurements. 

## 5. Conclusions

Low-cost sensors from state-of-art approaches were selected and their usefulness was verified for vehicle/pedestrian detection system applications. The sensors considered in this study do not enable detecting vehicles and pedestrians with high accuracy when used independently. The detection accuracy can be significantly improved by fusing data from an appropriately selected set of sensors. During the experiments reported in this paper various sensor sets were analyzed. The experimental results show that accurate vehicle detection can be achieved by using sets of passive sensors. For instance, the vehicles can be detected with 98% accuracy based of dataset containing magnetometer and light sensor readings. In case of pedestrian detection, application of active sensors (microwave radar, LIDAR) was necessary to obtain satisfactory results.

Vehicle and pedestrian localization tasks are also covered by the experimental evaluation presented in this paper as a supplementary function of the low-cost detectors. The objective was to recognize objects’ location with precision of 1 m using the same sensor node as for the detection experiments. The sensor node was installed on road side, in the center of the detection area. The localization accuracy achieved by single sensor node was below 75% for vehicles and 70% for persons. In order to improve the localization accuracy, it would be necessary to collect the measurements from more than one sensor node. Therefore, further research will be performed with wireless sensor network composed of multiple sensor nodes installed along the road. The sensor network is also expected to recognize direction and estimate speed of the traffic participants.

## Figures and Tables

**Table 1:** Usefulness of sensing technologies.

Sensing Technology	Principle of Operation	Requirements			
		Cost	Small Dimensions	Energy Consumption	Easy to Install
Inductive loops	Inductance measurement	Low	No	High	No
Cameras	Image analysis	High	Yes	High	Yes
Magnetometers	Magnetic field measurement	Low	Yes	Low	Yes
Acoustic sensors	Acoustic pressure measurement	Medium	Yes	Low	Yes
Radars/LIDARs	Detection of reflected electromagnetic wave	High	No	High	Yes
Accelerometers	Vibration measurement	Medium	Yes	Low	Yes
Light sensors	Light intensity measurement	Low	Yes	Low	Yes
Passive infrared sensors	Infrared radiation measurement	Medium	Yes	Low	Yes
Ultrasonic sensors	Detection of reflected sound wave	Low	No	Medium	Yes
Wireless communication devices	Measurement of received signal strength	Low	Yes	Medium	Yes

**Table 2:** Representative works related to low-cost traffic monitoring methods.

Study	Application	Data Source	Description	Important Findings	Comments
Mao et al., 2013 [7]	Object tracking	Wireless network with 40 light sensors	Trajectories and speeds of moving persons were estimated in indoor environment	Light sensor is sensitive to change of the light level in the environment even if multiple light sources are present.	Energy consumption is high as dedicated light sources are necessary.
Roy et al., 2011 [16]	Detection of road congestion	Wireless transmitter -receiver pair	Free-flowing and congested traffic states were recognized based on signal strength, link quality and packet reception metrics	Accuracy does not depend on transmitter power. Low accuracy is obtained when transmitter -receiver distance is short (<20 m).	Individual vehicles were not detected. Low-cost, energy efficient ZigBee modules were implemented.
Horvat et al., 2012 [15]	Detection of vehicles	Wireless transmitter -receiver pair	The possibility of detecting vehicles based on RSSI measurements was demonstrated	Pass of a vehicle causes drop of RSSI value. Gradient of the RSSI drop depends on vehicle velocity.	Accuracy of vehicle detection was not evaluated. Low-cost, energy efficient ZigBee modules were implemented.
Kassem et al., 2012 [17]	Vehicle detection and speed estimation	Wireless network with 2 transmitters and 2 receivers	Stopped and moving cars were recognized based on mean and variance of RSSI. Relation between RSSI variance and vehicle speed was used for speed estimation.	Presence of a vehicle affects mean of RSSI. The change of RSSI can be negative or positive. Variance of RSSI decreases when vehicle speed increases.	High accuracy of vehicle detection was achieved using a small dataset of 20 vehicles. WiFi devices were implemented that have high energy consumption.
Won et al., 2017 [13]	Vehicle detection, classification, speed estimation, and lane recognition	Wireless transmitter -receiver pair	CSI data were used to detect vehicles, categorize them as cars or trucks, estimate their speeds and recognize traffic lane.	Number of CSI samples collected while a vehicle passes between transmitter and receiver can be used for estimation of vehicle speed.Vehicles on different lanes exhibit distinct CSI distributions.	A large dataset was used for experiments (400 vehicles).WiFi devices were implemented that have high energy consumption.
Haferkamp et al., 2017 [14]	Vehicle detection and classification	Wireless network with 3 transmitters and 3 receivers	RSSI data were used to detected vehicles and categorize them as cars or trucks.	Ray tracing simulations are helpful in classifier training and optimization of antenna parameters.	High classification accuracy was achieved for a large test dataset.The use of directional antennas increases hardware cost.
Bernas et al., 2018 [20]	Vehicle detection and classification	Wireless network with 4 transmitters and 4 receivers	RSSI data were used to detected vehicles and categorize them as cars, semi trucks or trucks	Dependency exists between height at which devices are installed and the ability to detect particular vehicle class.	High classification accuracy and low energy consumption were achieved by using Bluetooth low energy modules.
Mrazovac et al., 2013 [22]	Human detection	Wireless network with 4 transceivers	Human presence was detected based on frequency analysis of RSSI variations.	Human presence can be detected and distinguished from background update via analysis of RSSI variations.	Experiments were conducted in indoor environment.
Hostettler et al., 2011 [25]	Vehicle detection	Accelerometer	Passing vehicles were detected in simulated and real-world scenarios	Adaptive threshold detection algorithm enables accurate vehicle detection based on road vibration measurements.	High accuracy was achieved using real traffic data for 142 vehicles. False detections can occur, e.g., due to nearby construction work.
Hostettler et al., 2012 [26]	Estimation of vehicle speed, wheelbase, and distance from road edge	Accelerometer	Speed of vehicle, its wheelbase, and distance from road edge was estimated by using extended Kalman filter.	Road surface vibrationsmeasured by single accelerometer can be used to track vehicles moving along a straight road.	The results are presented for two specific car models. Small dataset was used for testing. A number of parameters have to be predetermined.
Ma et al., 2014 [27]	Vehicle classification	Wireless sensor network with 6 accelerometers and 3 magnetometers	Vehicles were categorized into 3 classes based on axle count and spacing.	Accelerometers can be used for detecting axle locations. Magnetometers enable estimating vehicle speed and recognizing gaps between vehicles.	Sensors are installed in surface of traffic lane. Tire has to roll directly on top of at least one accelerometer. Short battery lifetime.
Rivas et al., 2017 [29]	Vehicle detection, recognition of driving direction, speed estimation	Sensor network with 4 accelerometers	Vehicles were detected with accuracy of 80%. Travel direction was recognized with 90% accuracy. Average speed measurement error was of 27%	Frequency range of street vibrations is between 250 Hz and 400 Hz. Sensors must have a range at least of 1 kHz.	Sensors mounted on roadside. Disadvantages are large size and high cost of sensors (>1000€ per unit). False detections are caused by bicycles.
Ghosh et al., 2015 [30]	Vehicle detection	Geophone and seismometer	Vehicles were detected in different scenarios: single vehicle moving, multiple vehicle moving, person walking and running nearby sensor.	The method is suitable for vehicle detection in domain of defense and perimeter monitoring.	The experiments were conducted for three types of vehicles: bus, tractor and truck. Personal cars were not considered.
Taghvaeeyan et al., 2014 [33]	Vehicle counting, classification, speed estimation	Four anisotropic magnetoresistive sensors	188 vehicles were detected and classified. Additionally, speed was estimated and right turns were recognized at intersection.	Speed of vehicle can be estimated based on cross-correlation between signals from two sensors. Classification can be performed based on magnetic length and magnetic height of vehicles.	Four vehicle classes were considered. Sensors are placed on road side. Precise time synchronization of sensors is required.
Balid et al., 2016 [34]	Vehicle detection and speed estimation	Wireless sensor network with two magnetometers	One sensor was used for vehicle detection.Speed was calculated based on travel time between twolongitudinally positioned sensor nodes	Variations of magnetic flux for sensors on road side are relativelyuniform when compared to sensors in traffic lane, which accounts for slightly better accuracy.	The system can be installed on surface of traffic lane or on road side.GPS modules were used for time synchronization.
Jo et al., 2014 [38]	Vehicle detection	Ultrasonic sensor	Single sensor was installed on road side to detect vehicles in two-lane road.	Ultrasonic sensors should only be used on roads with few lanes and moderate traffic volume.	Vehicle detection across multiple lanes with a single roadside ultrasonic sensor suffers a reduction in detection accuracy under dense traffic flow.
Volling 2013 [40]	Bicycle detection	Microwave radar	Tests with a small bicycle have confirmed high accuracy of the detection.	Simple analysis of sensor readings enables differentiating bicycles from vehicles.	Radar has to be installed in surface of the road.
Barbagli et al., 2011 [41]	Vehicle detection and speed estimation	Pair of microphones	Prototype was tested on a motorway. The collected data include vehicle counts and average speeds.	For vehicle speed above 30 km/h, the dominant sound sources are tires. For stopped vehicles, the dominant soundis motor noise. This fact can be used for traffic jam detection.	Sensor node is installed on the motorway’s guardrail and powered by rechargeable battery assisted by solar panel.
George et al., 2013 [44]	Vehicle detection and classification	Pair of microphones	Vehicles were detected based on peaks of low pass filtered acoustic energy. Neural network was used to categorize vehicles into 4 classes.	A peak finding algorithm for vehicles detection was proposed. Mel-frequency cepstral coefficients are useful for audio-based vehicle classification.	Low classification accuracy was achieved (67%). Multilane traffic was not considered. Impact of acceleration and gear shift on classification needs further exploration.
Na et al., 2015 [46]	Vehicle detection, classification, speed estimation, and lane recognition.	Array of 37 microphones	Vehicles were detected in 3 traffic lanes of a highway. Two vehicle classes were recognized based on time taken to pass detection zone.	Acceptable accuracy of vehicle countingand speed estimation can be achieved. High error rate was encountered for lane occupancy calculation and vehicle classification.	Microphone array has large dimensions and requires supporting structures.

**Table 3:** Advantages and limitations of low-cost sensing technologies for road traffic monitoring.

Sensing Technology	Advantages	Limitations
Infrared and visible light sensors	monitoring of multiple traffic lanes is possible enable pedestrian and bicycle detection detection range is wide	sensitive to light and weather variations cleaning is necessary
Signal strength analysis in wireless communication networks	robust against light and weather variations additional information can be transmitted monitoring of multiple traffic lanes is possible	devices have to be installed on both sides of the road, above road surface interference in ISM bands
Accelerometer applications	robust against light and weather variations enables wheelbase detection and counting	objects are not detected while not moving sensitive to vibrations in the environment
Magnetometer applications	robust against light and weather variations	sensor has to be installed inside or close to traffic lane unable to detect pedestrians/bicycles
Ultrasonic and microwave radars	robust against light and weather variations monitoring of multiple traffic lanes is possible provide speed information enable pedestrian and bicycle detection	wave-reflecting object has to be present on opposite side of the road Doppler sensors do not detect stopped objects
Acoustic sensing	robust against light and weather variations monitoring of multiple traffic lanes is possible	complex computations are necessary to eliminate impact of other sound sources

**Table 4:** Built-in sensors of SN1.

Sensor Type	Producer	Model	Sensitivity	Range
Accelerometer/gyroscope	Bosch	BMI160	16384 LBS/g	+/−2 g
Magnetometer	Yamaha	YAS537	0.3 μT	2000 μT
Light sensor	Liteon	LTR55X	0.6 lux	10,000 lux
Microphone	Xiaomi	-	-	40 Hz–48 kHz
Bluetooth module ^1^	Xiaomi	BLE 4.1	-	−100–0 dBm

^1^ used for measurement of RSSI.

**Table 5:** Sensors installed in SN2.

Model	Sensor Type	EnergyConsumption	Precision	Comments
SEN0158	Infrared camera	44 mA		Range 0–3 m
DFR0052	Piezoelectric vibration sensor	0	-	-
SEN-14032	LIDAR	130 mA	+/−25 cm	Range 0–40 m
GP2Y0A710K0F	Infrared distance measuring sensor	30 mA	-	Range 1–5.5 m
LOGO Sensor	Accelerometer	B/D	-	-
SEN-09198	Piezoelectric vibration sensor	0	+/−1%	-
MLX90614ESF	Infrared sensor	1 mA	+/−0.5 °C	-
7644 HB100	Microwave Doppler radar	30 mA	-	Range up to 20 m
7181 BH-1750	Light sensor	120 μA	20%	-
SEN0171	Passive infrared sensor	15 μA	-	7 m
BMP280	Barometer	2.7 μA	±0.12 hPa	-
HMC5883L	Magnetometer	100 μA	1°	-
ISL29125	Light sensor	56 μA	300 lux	-
LSM9DS1	Magnetometer, accelerometer, gyroscope	600 μA	1.13%	-
SEN0192	Microwave doppler radar	37 mA	-	-
HC-SR501	Passive infrared sensor	65 mA	-	-
JSN-SR04T	Ultrasonic distance sensor	30 mA	1 cm.	-
ADXL355Z	MEMS accelerometer	138 μA	±2 g–±8 g	-

**Table 6:** Accuracy of object detection based on individual sensor readings from SN1.

Sensor Reading	Object Detection Accuracy (%)		
	Vehicle	Pedestrian (Walk)	Pedestrian (Run)
Accelerometer x	46	64	49
Accelerometer y	46	63	57
Accelerometer z	58	58	57
Gyroscope x	40	53	70
Gyroscope y	44	49	59
Gyroscope z	49	49	34
Magnetometer x	93	49	52
Magnetometer y	61	47	52
Magnetometer z	83	42	48
Microphone	95	89	97
Light sensor	60	79	57
RSSI	85	42	43

**Table 7:** Accuracy of vehicle detection based on combined sensor readings from SN1.

Dataset	Algorithm	Aggregates	Vehicle Detection Accuracy (%)
Full	DT	average, standard deviation	100
		median, minimum, maximum	100
	KNN	average, standard deviation	100
		median, minimum, maximum	100
	MLP	average, standard deviation	92
		median, minimum, maximum	77
Without sound	DT	average, standard deviation	88
		median, minimum, maximum	85
	KNN	average, standard deviation	94
		median, minimum, maximum	94
	MLP	average, standard deviation	82
		median, minimum, maximum	85

**Table 8:** Accuracy of object detection based on individual sensors in SN2.

Sensor	Detection Accuracy (%)		Comments
	Vehicle	Pedestrian	
Accelerometer	60	64	Enables detection of heavy vehicles. Needs to be bonded to road surface.
Magnetometer	93	49	Detects vehicles in range of 2 m. Does not efficiently detect pedestrians.
Light sensor	60–95	60–85	Detects objects in range of 4 m. Sensitive to ambient lighting conditions and sensor orientation.
Passive infrared sensor	39	69	Low detection accuracy for both vehicles and pedestrians. Detection range limited to 1 m.
Infrared distance measuring sensor	80	76	Detection range up to 5 m. The object must cut the narrow beam otherwise will not be detected.
LIDAR	83	78	Detection range up to 5 m.
Piezoelectric vibration sensor	50	50	Low detection accuracy for both vehicles and pedestrians.
Barometer	52	50	Low detection accuracy for both vehicles and pedestrians. Detection range limited to 50cm.
Microwave Doppler radar (SEN0192)	76	69	Directed perpendicular to road axis. Detection range up to 5 m.
Microwave Doppler radar(7644 HB100) without housing	41	44	Oriented 45 degrees to road axis. Difficult to tune and configure.
Microwave Doppler radar(7644 HB100) with housing	81	79	Installed 2 m above a road/sidewalk.

**Table 9:** Accuracy of vehicle detection and localization based on combined sensor readings from SN2.

Sensors	Accuracy (%)	
	Localization	Detection
**Passive sensors**		
accelerometer, light sensor, vibration sensor, magnetometer, PIR, barometer	64	98
magnetometer, light sensor	69	98
accelerometer, vibration sensor, magnetometer, PIR, barometer	68	98
accelerometer, vibration sensor, magnetometer, PIR	68	98
accelerometer, vibration sensor, magnetometer	72	97
accelerometer, magnetometer, light sensor	71	98
**Active sensors**		
ultrasonic sensor, microwave radar, LIDAR, infrared camera	57	97
microwave radar, LIDAR, infrared camera	58	97
ultrasonic sensor, microwave radar, LIDAR	49	97
**Passive and active sensors**		
accelerometer, vibration sensor, magnetometer, microwave radar	73	98

**Table 10:** Accuracy of pedestrian detection and localization based on combined sensor readings from SN2.

Sensors	Accuracy (%)	
	Localization	Detection
**Passive sensors**		
accelerometer, light sensor, vibration sensor, magnetometer, PIR, barometer	58	84
accelerometer, light sensor, PIR	59	89
light sensor, PIR	60	88
**Active sensors**		
ultrasonic sensor, microwave radar, LIDAR, infrared camera	45	94
ultrasonic sensor, microwave radar, LIDAR	45	94
microwave radar, LIDAR	49	95
**Passive and active sensors**		
microwave radar, LIDAR, light sensor, PIR	66	95
